# Assembling a global database of malaria parasite prevalence for the Malaria Atlas Project

**DOI:** 10.1186/1475-2875-6-17

**Published:** 2007-02-16

**Authors:** Carlos A Guerra, Simon I Hay, Lorena S Lucioparedes, Priscilla W Gikandi, Andrew J Tatem, Abdisalan M Noor, Robert W Snow

**Affiliations:** 1 Spatial Ecology and Epidemiology Group, Department of Zoology, University of Oxford, Tinbergen Building, South Parks Road, Oxford, OX1 3PS, UK; 2 Malaria Public Health & Epidemiology Group, Centre for Geographic Medicine, KEMRI-Wellcome Trust-Collaborative Programme, Kenyatta National Hospital Grounds, P.O. Box 43640-00100 Nairobi, Kenya; 3 Centre for Tropical Medicine, John Radcliffe Hospital, University of Oxford, Oxford, OX3 9DS, UK

## Abstract

**Background:**

Open access to databases of information generated by the research community can synergize individual efforts and are epitomized by the genome mapping projects. Open source models for outputs of scientific research funded by tax-payers and charities are becoming the norm. This has yet to be extended to malaria epidemiology and control.

**Methods:**

The exhaustive searches  and assembly process for a global database of malaria parasite  prevalence as part of the Malaria Atlas Project (MAP) are described. The different data sources visited and how productive these were in terms of availability of parasite rate (PR) data are presented, followed by a description of the methods used to assemble a relational database and an associated geographic information system. The challenges facing spatial data assembly from varied sources are described in an effort to help inform similar future applications.

**Results:**

At the time of writing, the MAP database held 3,351 spatially independent PR estimates from community surveys conducted since 1985. These include 3,036 *Plasmodium falciparum *and 1,347 *Plasmodium vivax *estimates in 74 countries derived from 671 primary sources. More than half of these data represent malaria prevalence after the year 2000.

**Conclusion:**

This database will help refine maps of the global spatial limits of malaria and be the foundation for the development of global malaria endemicity models as part of MAP. A widespread application of these maps is envisaged. The data compiled and the products generated by MAP are planned to be released in June 2009 to facilitate a more informed approach to global malaria control.

## Background

In an era dominated by information, the need to digest research findings into public domain data repositories is key to enhancing scientific enquiry [1]. The approach to characterizing the human genome through assemblies of research by numerous groups across the world represented a watershed model for information sharing and accelerated discovery across the life-sciences [2-5]. This philosophy of public access in genetic research has advanced our basic understanding of the malaria parasites [6-9] and the *Anopheles gambiae *vector [10,11]. Public access models have also been applied to quality assured biological standards and standard operating procedures for laboratory methods [12], the establishment of registers of contemporary protocols for Phase III clinical trials [13] and a broader, donor-led movement, to ensure that findings of funded medical research are published in the open access literature [14-16].

The design, financing and implementation of malaria control demand a quantitative approach to the definition of anti-malarial commodity requirements of populations living under varied malaria transmission risks. The geographic extent of effective intervention coverage and how this relates to the dominant local *Anopheles *vector species and, in turn, their sensitivity to insecticides, is necessary to design appropriate vector control. Information on the prevalence of drug resistance and population spatial access to medicines is required to devise adequate clinical disease management strategies.

The Mapping Malaria Risk in Africa (MARA) [17] collaboration was launched over a decade ago to help provide a geo-positioned series of malariometric data for Africa [18]. MARA succeeded in collating a large private database of independent PR surveys across the continent by the year 2000. The data generated has been used as the basis for many innovative modelling and mapping efforts in Africa [19-26]. A continent-wide map of malaria transmission intensity, however, has not been published and, more importantly, the empirical data on parasite prevalence has not been made available for public access.

The recently launched Global Health Atlas of the World Health Organization (WHO) [27] aims to provide malaria-related data available to the public. However, the fidelity of the information managed by WHO has been questioned [28]. The database includes misleading entries, such as those for Kenya and Somalia that report only 135 and ten malaria deaths in 2002, respectively [29]. Less than half (22/49) of the malaria endemic countries (MECs) in Africa provided information for the most recent reporting year, 2003; the rest were older. These data are neither reconciled at sub-national scales, nor against populations at risk of any standard definition of the intensity of malaria transmission.

Recent work has also involved the assemblage of historical and contemporary evidence on the distributions of malaria risk and *Anopheles *vector species into a compendium of maps and geographic descriptions of various malaria indicators by region and country [30]. This is a significant contribution to medical intelligence on the global spatial epidemiology of malaria, but lacks consistency in the sources of information used to generate the maps and is not structured on the foundations of a systematic search and archive of relevant information in a geo-positioned database. In addition, local efforts to improve spatial information relevant to malaria, while impressive (see, for example, [31] and [32]) do not have a global coverage.

More recently, plans have also been articulated for the construction of a global database on anti-malarial drug resistance [33]. This database aims to include current and historic data on clinical efficacy, *in vitro *responses of patient isolates to old and new antimalarial drugs, as well as molecular markers of drug resistance in *P. falciparum *and *P. vivax *malaria [33]. This initiative was stimulated by the fact that, despite being theoretically available, information is scattered in publications and across institutions, which makes it relatively difficult to assemble.

Information resources on malaria risk, disease incidence, intervention coverage or drug resistance should have several key features to maximize their utility for constituents. Data assembly requires a filtering system in which data inclusion rules are clearly defined and some basic standards are set before archiving. These inclusion criteria and any methods used during data collation and data entry need to be scientifically defensible and transparent. Finally, and crucially, databases need to be in the public domain in order to promote the advancement of science and its equitable distribution among contributors.

Despite the unprecedented expansion in international financing of malaria control worldwide [34], the distribution of this investment has not been guided by any clear epidemiological or population-at-risk based criteria. One obvious reason has been the lack of a scientifically credible, globally assembled, epidemiological information platform to guide decision-making. It is not that relevant epidemiological data do not exist, rather that they have not been: a) assembled from their diverse sources into a single data repository; b) used in an informed, scientifically robust way to resolve global malaria risks spatially; or c) made available in the public domain. The recently launched Malaria Atlas Project (MAP) aims to fill this role [28,35].

One of the main goals of MAP is to re-assemble contemporary information on the limits and risk of malaria infection across the 106 current MECs [36,37]. When attempting to describe and ultimately map malaria endemicity globally, a unifying variable representing malaria risk must be chosen. The characteristics of such a descriptor include a global scope, a high frequency of standardized sampling, a clear link to the abiotic factors that determine the ecological foundations of malaria risk and a useful representation of the variations in age-specific malaria morbidity and mortality rates. The most commonly recorded measure of malaria risk remains the parasite rate (PR). PR surveys are more frequently reported in bibliographic archives than all of the other epidemiological metrics combined (Table 1). It was, therefore, justified to focus the initial information searches of MAP on the retrieval and archive of PR surveys undertaken across MECs. This paper presents the methods and approaches used to assemble a global database on this key measure of malaria endemicity and risk. It is also a comprehensive documentation and citation source for the data that are destined to become freely available in the public domain in June 2009.

**Table 1:** Frequency of malaria transmission indices in two bibliographic archives.

Index	PubMed hits	ISI Web of Science
Entomological inoculation rate	97	84
Vectorial capacity	120	97
Basic reproductive number	10	7
Parasite rate	889	616

The search terms used were 'malaria' and each index as it appears in the table. Searches were run on February 2007.

## Methods

The MAP collaboration has adopted three linked approaches to identifying empirical PR survey data: a) a traditional electronic search using PubMed [38] with 'malaria' and MEC name as free text rather than Medical Subject Headings terms that tend to be less inclusive; b) direct contact with malaria field scientists, research institutions and control agencies in MECs identified through the PubMed search; and c) an e-mail circular, linked to the launch of the MAP website, to locate sources of information not readily accessible from the first two search strategies.

A number of inclusion rules were imposed for information identified through the multiple searches (see Additional File 1). These rules have been implemented to ensure spatial independence of data, precision in individual estimates, standardization of parasite detection methods, and avoidance of confounding effects through malaria specific interventions. The aim was also to collect the most contemporary data possible. During the searches, a review of titles and abstracts was used to eliminate sources that did not match the inclusion criteria.

Authors of peer-reviewed sources of PR survey data were contacted if: a) additional information was required on the age-ranges; b) multiple community data needed to be disaggregated; or c) specific details on the coordinates or location of the survey data were needed. Additionally, authors were asked if they knew of other unpublished information on parasite surveys undertaken in their country of research. This request was extended to over 100 institutions involved in malaria research and control identified as potentially useful sources of information during the formal literature search. These included, amongst others, the Environmental Health Project (EHP) of USAID, Médecins Sans Frontières (MSF), Merlin, UNICEF, WHO regional offices, as well as, national institutes of research in China, India, Kenya, Tanzania, Thailand, and other countries. This second-line search strategy aimed at identifying 'grey' literature sources (publications issued by government, academia, business and industry not controlled by commercial publishing interests) and primary, unpublished PR survey data.

Finally, the database of malaria research scientists and people involved in malaria control was expanded from the authors and institutions lists in the literature, malaria research conference and meeting attendance lists, and membership listings published by national and international tropical medicine societies. The database includes contact details and the MEC of professional affiliation. This database provided the basis for the third approach to identifying PR survey data: sending an e-mail circular alerting recipients to the launch of the MAP initiative, indicating the data needs of the project and directing them to the MAP website [35]. The website was constructed to list country-specific data already abstracted and highlight, through on-line maps, areas where data are currently unavailable and where their provision would assist in the coverage of information within national boundaries.

### Assembling a digital data archive

Each source of information was reviewed by one of the authors of this paper and the data extracted into a customized Microsoft Access (Microsoft, 2003) database. The main data entry form displays information of three different types. First, it allows entry of basic information related to the data source. A unique, auto-generated identifier links the record to a reference manager platform and to an electronic copy of the source when this could be obtained. Paper copies of published or unpublished reports not available in an electronic format were scanned and converted to portable document format (PDF). The design of the digital library is one of the main strengths of the MAP database as it allows an easy, rapid and organized access to original sources for data verification and follow-up. It may also present a future weakness for data sharing since many of the literature are protected by copyright.

The entry form includes all fields related directly to malaria prevalence, including some geographic descriptions (geographic extent of the study area, as well as the land cover type as reported by the author(s) as either urban or rural, and forest and/or rice cultivation), and a full description of the cross-sectional study and its results (number of surveys, parasite detection method, dates, age-range sampled, number of slides examined and numbers of positive individuals). The contacts database is also a crucial component of the MAP main database. The form is linked to an email directory to help keep track of all communications with individuals as separate text files enabling an electronic trail of correspondence to re-check information and avoid duplication of messages to individuals by different members of the MAP team.

A geo-positioning entry form was designed separately that links directly to the relevant section of the main Access form. In this way, locations and geo-positioning can be updated independently without affecting the main PR data table. This form shows descriptions of the location in which the survey was conducted. These include the name of the community studied and its regional, country and sub-national correspondence, as well as the geographic coordinates and related information about the geo-positioning method. Geographic data entry works on the basis of drop-down menus based on country and sub-national first (ADMIN1) and second (ADMIN2) administrative division tables derived from the United Nations' Second Administrative Level Boundaries (SALB) dataset [39] and the Global Administrative Unit Layers (GAUL) developed by the Food and Agriculture Organization (FAO) [40]. This allows the user to constrain the search progressively by administrative division and decide on the best match for each site accordingly. By doing so, the user also assigns geographically unique codes to the location automatically, in a hierarchical fashion (*i.e*. ADMIN2 codes are linked to respective ADMIN1 codes and these to country codes). Thus, each community is assigned ultimately a unique identifier that, not only records its geographic coordinates, but ascribes the site to the correct administrative units on a map.

The main database is linked to a geographic information system (GIS). This, coupled with queries designed to generate custom tables to display data according to region, country, PR data source, parasite species and other custom criteria, allows a simple and rapid generation of the country and regional maps found on the MAP website. This information is updated on a weekly basis.

### Locating survey data

The geo-positioning of surveyed communities is a complicated exercise. There are a number of potential problems when locating a survey in space and the level of difficulty depends largely on the amount of information available for the survey area in question. Having only the name of the location is often insufficient because village names are frequently repeated within countries and even within administrative sub-divisions. Also, Anglicized spellings may vary with translation and are complicated by the diversity of alphabets, sometimes making sites unidentifiable, despite being clearly available on a map. Although standard nomenclatures and rules for translation are attempted in digital gazetteers [41] these are difficult to achieve at national levels, let alone globally. Every data point was, therefore, recorded with as much geographic information from the source as possible and this was used as an aid during the searches and geo-positioning. When available, this information included its first, second and third administrative division associations, as well as any useful landmarks close to the site that could be used to locate it on a map (*e.g*. distance from, or relative location with respect to, a main city or an obvious geographic feature).

**Figure 1:**
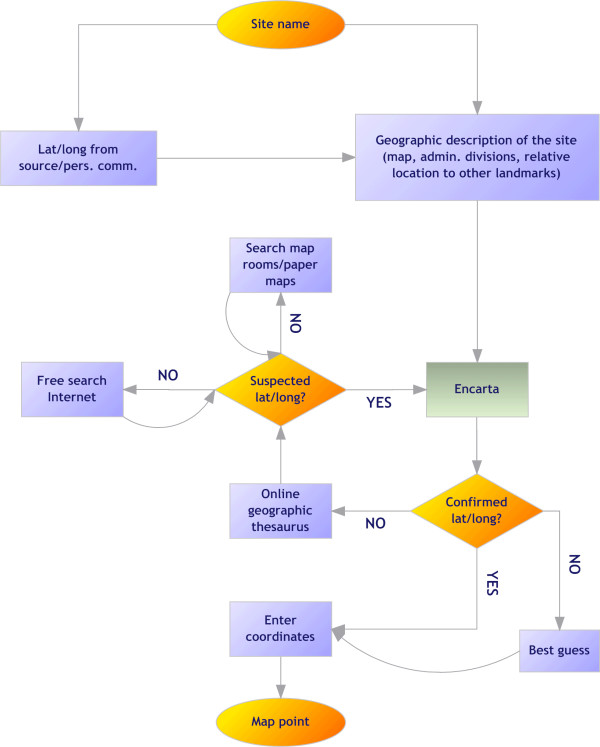
Flow chart for the geo-positioning of PR data points.

A flow chart of the geo-positioning process that illustrates the hierarchy of decisions made is shown in Figure 1. All geographic coordinates were standardized to decimal degrees in order to be displayed in a geographic projection, which was preferred given its wide use and support. The Encarta Encyclopaedia (Microsoft, 2007) was used as the standard for geo-positioning due to its comprehensive database of geographic names and its dynamic interface. Navigating in this way helps relate the point searched with neighbouring geographic features and, therefore, geo-position the place with more accuracy. If a site was not found in Encarta, priority was given to coordinates obtained directly from the source, provided these were given with precision (*i.e*. defined at no less than two decimal places considering that 0.0083 degrees is the approximate equivalent to 1 km at the equator). Accurate geographic coordinates obtained through personal communication with the authors, or people knowledgeable of the area, were given the same priority. When this information was not available, other electronic sources were used (*e.g*. Africa Data Dissemination Service [42], Alexandria Digital Library [43], EC Joint Research Centre Digital Atlas [44], Falling Rain Genomics Inc.'s Global Gazetteer [45], GEOnet Names Server [46], Getty Thesaurus of Geographic Names [47], Google Earth [48], Maplandia [49]). These sources are available freely on-line and provide varying degrees of coverage, functionality and ease of use. If these proved unsuccessful, then searches both in Google [50] and in printed documents (*i.e*. paper maps and gazetteers in map rooms) were undertaken. It is worth noting that the latter yielded disappointing results, with no more information than was available from on-line sources. All promising matches were then again mapped in Encarta to confirm accuracy, before accepting the location as a valid data point. The last resort was to attempt a 'best guess' of where the point is located. The most common example of this approach was when an inaccurate map was available from the source, usually only for illustrative purposes. This was used to derive geographic coordinates by extrapolating the information to Encarta and approximating the position. Detailed notes of the decisions made during geo-positioning have been kept.

It is not always clear how communities are described in space during PR surveys. In East Africa, it is common to define a community as represented by widely dispersed homesteads, separated sometimes by several hundreds of metres of agricultural land. Conversely, in several West African countries, a community is more spatially constrained with agricultural land surrounding the village unit. PR survey data are reported by community name and generally not spatial dimensions or settlement density. Thus, any decisions on positioning survey data are, to some extent, arbitrary. Another common description of communities presented in survey sources are small collections of neighbouring villages. In such cases, authors were often contacted to provide specific information for each village. Where these data were not available, it sometimes seemed reasonable to assume that these represented a single community. In all cases, notes identifying the decision taken for a particular sample were recorded.

The following rules were implemented in attributing data as representing a 'point', a 'wide-area' or a small or large 'polygon'. A point was considered as such when the community was named specifically and could be located in space unambiguously, or when the information suggested that a study area was no bigger than ~10 km^2^. A wide-area was defined as any study area that exceeded ~10 km^2 ^but was no bigger than ~25 km^2^. A small polygon was assigned to an area between ~25 km^2 ^and ~100 km^2^. All areas for which information suggested that they were larger than this value were defined as large polygons. Inevitably, these decisions were crude but informed by the knowledge of the problems that coarse spatial occurrence data presents for developing high spatial resolution models of risk [51]. The thresholds were determined largely by the resolution of the ancillary data compiled as part of MAP, which will be described elsewhere, and is standardized mainly to 1 km^2^. Although it would be highly desirable to expect PR data at this level of resolution to qualify as a point, such a definition would be often impractical and regularly illusory. The 10 km^2 ^point tag is pragmatic because it avoids the exclusion of large quantities of data while allowing for some of the obvious uncertainty inherent during geo-positioning. It is likely that wide-areas and polygons will be excluded from some of the later analyses, particularly those that involve ancillary data in which a high level of spatial variation is expected (*e.g*. population density, altitude, forest cover). Wide-areas and polygons in the MAP database include groups of villages for which a single PR estimate was available, small islands and high resolution administrative units (ADMIN3 or higher). Whenever possible, polygons were converted to wide-areas by using assumptions based on population distribution within a large area.

## Results

### Search strategy

The data search began in March 2005 with a focus on Africa and was extended to a global search in July of the same year. The PubMed search strategy was repeated twice before the end of October 2006. In total, 13,709 PubMed hits were returned for the 106 MECs [36,37] for the period between 1985 and 2006. This first search strategy yielded a total of 425 peer-reviewed publications containing data that fulfilled the inclusion criteria shown in Additional file: 1. These sources represented 63.3% of the total sources used in the MAP databases. As part of the second search strategy, a total of 306 different people were contacted directly for additional information to support the extraction of data from peer-reviewed publications or who were thought to have primary unpublished information. On 8 June 2006, 3,550 people were approached with a mass e-mail communication as part of the third data collection strategy using the extended contacts database. Since this database was compiled from a number of different sources, it was inevitable to find that some of the contact information would be outdated. Therefore, 1247 (35.1%) emails did not reach their destination. Unfortunately, only 19 replies to this communication were received, of which four provided actual data or leads to useful sources of additional data. Although this exercise was less productive than expected as a means of gathering data, it did increase the awareness of MAP and opened networks of communication that were previously unexplored, particularly in Latin America. Also, as part of this public engagement strategy, the MAP website was launched in May 2006. Between its launch and 31 October 2006, the MAP website received 2,079 unique visits from 115 different countries.

By 31 October 2006, a total of 671 primary sources of information were obtained through the three search strategies outlined. These sources yielded 3,680 records of community-based PR. For *P. falciparum *PR, 3,361 individual estimates were obtained from 664 sources and ranged from one to 219 records per source. For *P. vivax*, a total of 1,592 independent records were obtained from 283 sources, ranging from one to 269 per source. For most records (2,306), a single source was sufficient to extract all the data required. For the remainder, up to three sources were used to describe age, time and geographic specific information, which often involved direct communication with authors or scrutiny of complementary sources.

### Sources of information

Information sources were classified as follows: a) where data were reported as part of a survey aggregated across wide areas, and were subsequently refined to individual communities through correspondence with authors, the primary source was regarded as 'unpublished work' and the secondary source as 'journal', 'report', 'MoH (Ministry of Health) report', 'conference abstract', or 'thesis'; b) where only the geographic coordinates or age stratification were obtained through personal communication, but all other information was available from the original work, the primary source was classified as 'journal', 'report', 'MoH report', 'conference abstract', or 'thesis' and the secondary source entered as 'unpublished work'; c) where combinations of more than two source types were used to derive additional information for that record, the source that provided with the highest spatial, temporal and/or age stratification data was defined as the primary source, and the other two as secondary and tertiary sources.

Table 2 shows the primary, secondary and tertiary source combinations for the 3,680 records identified in total and by species. The most successful approach to identifying community specific estimates of parasite prevalence was through communications and contacts with malaria scientists identified from peer-reviewed literature sources. Assembling a comprehensive database of PR data cannot depend entirely on information available from peer-reviewed sources, therefore, as sources classified as 'journal' alone only provided 26.4% of the total number of records. This represents a generic point about database assemblies and demands a considerable effort from database gatekeepers and developers.

**Table 2:** Number of total *P. falciparum *and *P. vivax *records by type of source. The different combinations are described in the text.

	Records	Percent	Records *Pf*	Percent *Pf*	Records *Pv*	Percent *Pv*
Journal	972	26.4	965	28.7	487	30.6
Unpublished	727	19.8	687	20.4	352	22.1
Report	253	6.9	253	7.5	55	3.5
MoH Report	217	5.9	217	6.5	3	0.2
Thesis	106	2.9	106	3.2	48	3.0
Conference abstract	25	0.7	25	0.7	8	0.5
Other*	6	0.2	6	0.2	0	0.0
Journal + unpublished	50	1.4	50	1.5	22	1.4
Unpublished + journal	718	19.5	446	13.3	559	35.1
Report + unpublished	10	0.3	10	0.3	8	0.5
Unpublished + report	24	0.7	24	0.7	3	0.2
Unpublished + MoH	219	6.0	219	6.5	0	0.0
Thesis + unpublished	21	0.6	21	0.6	1	0.1
Unpublished + thesis	33	0.9	33	1.0	27	1.7
Unpublished + thesis + journal	3	0.1	3	0.1	3	0.2
Unpublished + report + journal	209	5.7	209	6.2	0	0.0
Unpublished + MoH + journal	40	1.1	40	1.2	0	0.0
Other combinations	47	1.3	47	1.4	16	1.0
Total	3,680	100	3,361	100	1,592	100

*Sources corresponding to un-referenced notes of institutions that could not be categorized as a full report.

Non-peer-reviewed reports by research agencies, NGOs and ministries of health are a potentially very useful source of information. The current MAP database contains 470 records from sources classified as primary, including 217 ministries of health reports from Kenya (89), Uganda (32), Mozambique (26), Swaziland (22), Tanzania (12) and Togo (11). This is an opportunistic means of data gathering and depends largely on the contacts available to those assembling the database, as evidenced by the strong links of MAP researchers within Africa. In addition, despite contacting 253 individuals known to be affiliated directly with ministries of health in other MECs, no returns were made by the 31 October 2006. Obviously, this does not mean that the data do not exist, but rather that they are more difficult to access than published data and more creative ways of retrieving them are required.

As part of 'grey' literature searches, an extensive exploration of post-graduate theses was undertaken. These proved a valuable resource by contributing with 106 PR records to the database. In addition, all large tropical medicine conference proceedings from 1995 were reviewed. Prior to this date, possible sources of information from international conference proceedings were only sourced when abstracts were easily available. This source of information resulted in only 25 PR records. Smaller national conference proceedings might have yielded more comprehensive data, but these were not easily accessible with the exception of one conference proceeding in India, available from the National Institute of Malaria Research website [52]. By the very nature of national conferences, post-graduate theses, 'grey' literature reports and ministry of health survey data, these will not have been captured comprehensively by the MAP data searches. It was the intention of the e-mail circular to request PR data from these sources, but this effort produced few records.

### Geo-positioning survey data

Of the 3,680 records, 1,565 were able to be geo-positioned either because authors provided detailed geographic coordinates in the report or they were obtained through personal communication. Of the remaining PR records, Encarta alone was useful in geo-positioning 897. Another 1,033 records were geo-positioned with a combination of web-based electronic gazetteers, illustrative maps in the source, free searches on the Internet and best guesses.

Geo-positioning was a labour intensive exercise, often involving multiple communications with authors. Inevitably, there were a few communities that were not possible to locate in space from any source (n = 185). Overall, 3,165 geo-positioned records were considered points, 106 wide-areas, 113 small polygons and 111 qualified as large polygons. The latter will be excluded from most analyses and are not considered here. A total of 296 data records (8% of the initial PR records retrieved) were, therefore, excluded. The total number of PR surveys geo-positioned successfully and excluding large polygon records was 3,384.

### Parasite detection and sample size

Of these 3,384 geo-positioned records, the PR was estimated using microscopy in 2,764 (81.7%) and using Rapid Diagnostic Tests (RDT) in 587 (17.3%). One large survey in Ghana, representing 31 communities, and a survey in Papua New Guinea, covering two communities, used PCR as their parasite detection tool. These were excluded from subsequent analyses given the higher sensitivity and lack of direct comparability with either microscopy or RDT. The ease of RDT methods means that these are increasingly used as part of large PR surveys (*e.g*. Cambodia, Equatorial Guinea, Kenya, Somalia, Tanzania, Thailand, and Zimbabwe). With time, these data will hopefully become more readily available and accessible to MAP.

The sample sizes of the PR records are a result of direct extraction from the source, an aggregation of information for a single community from more than one source, or through correspondence with authors. The database of geo-located point survey data described by microscopy or RDT contains an aggregated sample size of 1,500,203 examinations for *P. falciparum *infection globally and 895,278 examinations for *P. vivax*. Over 66% of all surveys included at least 100 examinations for either species, falling well within the prescribed ranges of precision required for parasite surveys [53].

### Survey time periods

The data have been structured according to four time periods: 1985–1989, 1990–1994, 1995–1999 and 2000–2006. The frequency of *P. falciparum *and *P. vivax *records reported during these periods is shown in Table 3, globally and by WHO region. More than half of the *P. falciparum *and *P. vivax *PR surveys covered the period beyond 01 January 2000 (53.2 and 59.2%, respectively) and the great majority were undertaken since 1995 (70.3 and 75.2%, respectively). In Africa, more than half of MAP *P. falciparum *PR records (51.4%) derive from surveys undertaken between 2000 and 2006. The year 2000 is the last for which MARA reports any data (n = 124) in their MARALite database [17]. The MAP dataset is, therefore, the only current reflection of *P. falciparum *parasite prevalence at the global scale, and the most contemporary in Africa. A similar pattern is reflected for *P. vivax*.

**Table 3:** Frequency of PR records by time period and WHO region.

WHO region	AFRO		AMRO		EMRO		EURO		SEARO		WPRO		Globe	
Period/Species	*Pf*	*Pv*	*Pf*	*Pv*	*Pf*	*Pv*	*Pf*	*Pv*	*Pf*	*Pv*	*Pf*	*Pv*	*Pf*	*Pv*

85–89	233	9	27	26	52	37	0	0	32	27	28	48	372	147
90–94	352	7	28	28	23	21	0	0	68	64	59	67	530	187
95–99	294	33	57	57	45	22	0	0	58	49	65	54	519	215
00–06	929	15	22	22	190	349	5	8	203	199	266	205	1,615	798
Total	1,808	64	134	133	310	429	5	8	361	339	418	374	3,036	1,347

*Pf *-*P. falciparum*,* Pv *-*P. vivax*, AFRO – African Regional Office, AMRO – Americas Regional Office, EMRO – Eastern Mediterranean Regional Office, EURO – European Regional Office, SEARO – South East Asian Regional Office, WPRO – Western Pacific Regional Office.

### Spatial distribution of PR records

**Figure 2:**
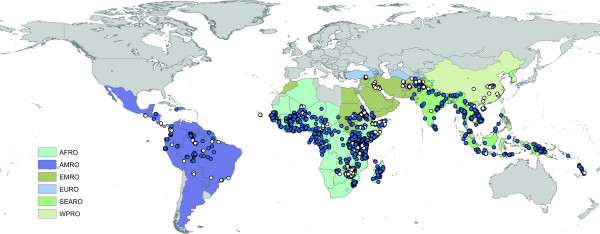
The global distribution of *P. falciparum *PR points from the MAP database. MECs are coloured by the WHO regional office to which they belong. Refer to the legend of Table 3 for abbreviations. The blue dots indicate presence (PR > 0) and white dots absence (PR = 0).

**Figure 3:**
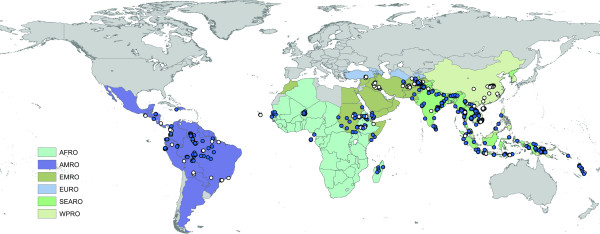
The global distribution of *P. vivax *PR points from the MAP database. MECs are coloured by the WHO regional office to which they belong. Refer to the legend of Table 3 for abbreviations. The blue dots indicate presence (PR > 0) and white dots absence (PR = 0).

**Figure 4:**
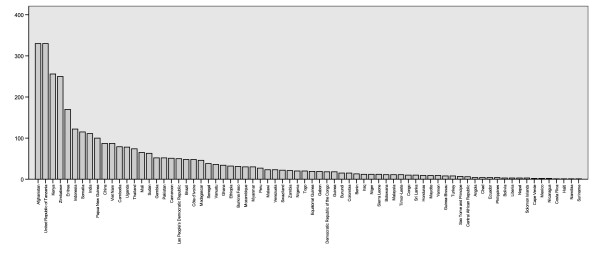
The number of PR records retrieved by country.

**Figure 5:**
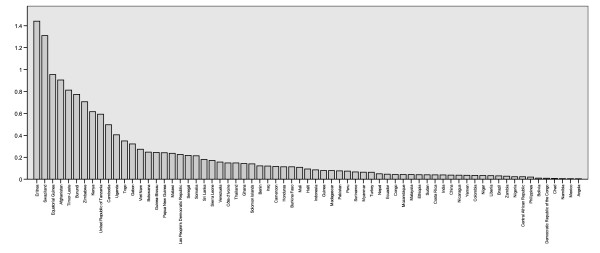
The number of PR records retrieved by malarious area by country. The scale expresses the number of PR surveys for every 1,000 km^2 ^of area malarious. The latter is as determined by the spatial limits of malaria in each country [36, 37]. Mayotte, Sao Tome and Principe, Gambia, Vanuatu and Cape Verde were excluded for visualization purposes because their very small malarious areas biased these calculations. These countries ranked top in the order listed.

The number of records varies substantially amongst regions and countries, reflecting not necessarily where malaria is a more serious problem, but where more PR estimates are available, either because surveys are conducted more systematically or because data proved easier to access. Endemic countries for which no data or no high spatial resolution surveys were available are Algeria, Argentina, Armenia, Azerbaijan, Bangladesh, Belize, Bhutan, Comoros, Djibouti, Dominican Republic, Egypt, El Salvador, French Guiana, Georgia, Guatemala, Guyana, Iran, Kyrgyzstan, Mauritania, Mauritius, Morocco, North Korea, Oman, Panama, Paraguay, Rwanda, South Africa, Saudi Arabia, South Korea, Syria, Tajikistan and Turkmenistan. After excluding PCR surveys, the MAP database holds 3,351 independent community PR estimates, including 3,036 *P. falciparum *and 1,347 *P. vivax *estimates. The global distribution of PR records is presented in Figures 2 and 3. Data for *P. falciparum *and *P. vivax *were retrieved for 74 and 41 countries, respectively. Figure 4 ranks these MEC by the number of records gathered. Afghanistan, Tanzania, Kenya, Zimbabwe and Eritrea are the top five countries in terms of number of PR survey records. A more balanced picture of data availability by country was produced by weighting the number of points by the estimated area that is malarious derived from the global spatial limits of malaria (Figure 5) [36,37]. According to this ranking, the five countries for which the least PR records are available are Angola, Mexico, Namibia, Chad and Democratic Republic of the Congo.

## Discussion

The last attempt to map the global endemicity of malaria was undertaken almost 40 years ago and was based largely on expert opinion and a mixture of historical evidence, climate and survey data [54,55]. The MAP database has, for the first time, gathered a large global set of spatially unique, contemporary empirical data on malaria endemicity. This paper has described in detail the methods used to compile these data and serves as the documentation necessary to interpret the database. This assembly of close to 3,500 estimates of parasite prevalence provides a very substantial step in generating the foundations for global malaria endemicity mapping within refined spatial limits [36,37] using modern techniques with high fidelity environmental data. A general overview of the procedures outlined to achieve this can be found elsewhere [28].

The development of the MAP PR database faced difficulties related to dealing with meta-data that can be considered generic to the assembly of large health spatial databases. It is clear from the methods reviewed here that such data collection cannot rely solely on published information as only one quarter of our PR data were available from sources found in the scientific literature. Although non-trivial, accessing the vast amounts of information in grey literature and unpublished sources is fundamental to maximizing information coverage. MAP has succeeded in procuring large amounts of data from these sources in some countries but not in others. In addition to a great number of individual malaria scientists, national and international institutions, including regional offices of the WHO (*e. g*. EMRO and SEARO), have proved highly responsive to requests for data. Moreover, MAP is actively cultivating collaborative links with relevant institutions in order to foster reciprocal data flows and sustainable long term support to the primary data gathering required to map malaria endemicity. It is hoped that the transparency of this exercise will stimulate further active data sharing in the future and expand the collaboration network of MAP.

Another significant obstacle confronted during the assembly of the MAP database was the geo-positioning of data. The accuracy of spatial references in a database is wholly dependent on the precision with which data are located in space. This problem was minimized by developing an elaborate system of geo-positioning based on using multiple sources of information and rigorous quality control. Strict definitions of the geographic nature of the study areas were set (*i.e*. point, wide-area, small or large polygon) in order to increase the accuracy of the spatial attribution of the PR data and control the inclusion or exclusion of records during analyses, depending on the spatial resolution of covariates. Useful tools for geo-positioning were identified and it is expected that new resources, such as Google Earth [48], will both facilitate and improve the accuracy of these tasks in the near future. This process was not without major difficulties and this methodology represents a substantial contribution for health geographic databases development. It is hoped that the wider use of global positioning systems to record the geographic coordinates of survey locations will decrease these problems.

MAP is also currently unique in that it represents one of the first attempts to make epidemiological data available in the public domain. This attempt requires detailed peer-reviewed documentation of the data search, assembly, geo-positioning and archival rubrics, and this is the main goal of this paper. The date for public release of these accumulated data was set as 01 June 2009. This time interval serves three main purposes: a) it allows sufficient time for MAP to be recognized as a collaboration of stability and longevity; b) it provides a realistic time-frame in which quality control, age-standardization and appropriate descriptions of the PR data can be achieved before release, and, importantly; c) it allows data sharing agreements to be negotiated with all data contributors. Providing the provenance of the PR survey data, through access to all supporting source PDF documentation, is an important aspect of this effort and, therefore, the viability of achieving this without infringing copyrights is being assessed.

MAP is committed to the continual improvement of the spatial coverage of the PR data. The availability of survey points, presented here by country, is sufficient for a general overview of progress, but subject to the environmental arbitrariness of national boundaries. The use of ecological distance metrics [56] would provide a more objective way to prioritize future search efforts within and between environmentally coherent zones. Since local *Anopheles *vectors can substantially affect local malaria endemicity within such ecozones, the MAP database is being augmented with vector species distribution records, so that these environmental distance metrics can be made vector specific. These vector distribution data will also enhance the ability to refine the global spatial limits of malaria [36,37], map global malaria endemicity and, ultimately, provide the basis of an informed approach to intervention and control. The ongoing effort to generate, archive and document information for these species globally will be described separately. Once robust spatial estimates of global malaria endemicity have been developed, the integration of further epidemiological data to exploit the applicability of these maps is anticipated, including monitoring progress towards international malaria control targets and the projection of financial and commodity needs for MECs. Without publicly available information, national and international agencies will continue to assume that entire regions or countries share similar epidemiological characteristics and that they must share one prescribed menu for control. The future success of malaria control at a global scale demands an investment in the assembly of epidemiological intelligence with a documented provenance. MAP is an attempt to fill this niche.

## Authors' contributions

CAG, SIH and RWS wrote the paper jointly. CAG designed and developed the MAP database. CAG, RWS and SIH implemented and supervised data searches, data entry and geo-positioning. LSL and CAG designed and developed the MAP website. LSL, PG and AMN supported data entry and geo-positioning. AJT provided support on ancillary data compilation.

## Supplementary Material

Additional file 1 The MAP database PR inclusion criteria Click here for file
