# Supplementary material for: Assembling a global database of malaria parasite prevalence for the Malaria Atlas Project
Source: Malar J. 2007 Feb 16;6:17. doi: 10.1186/1475-2875-6-17 (PMC1805762; doi:10.1186/1475-2875-6-17)
Supplement: Additional file 1 — The MAP database PR inclusion criteria [file 1475-2875-6-17-s1.pdf]

The MAP database PR inclusion criteria

Many factors can introduce bias when estimating the malaria PR of human populations. These include, amongst others, the age-range of the subjects investigated, short- and long-term temporal variations in transmission levels (*i.e.* seasonality, inter-annual variability and secularity) [1-4], heterogeneity of the host, vector and parasite populations [5, 6], and the immunity status of the sampled population [7-9]. More generically, the precision of the estimate depends both on the size of the sample and the underlying true prevalence of malaria [10]. A series of *a priori* inclusion criteria were defined and used during data collection to try to control for this bias. These criteria are classified as either strict (surveys were excluded if they did not comply) or preferred (did not necessarily exclude surveys but guided selection to the best data available).

Strict criteria	
Time of survey	The last 20 years were considered a balanced, yet admittedly arbitrary, period for PR data inclusion. All surveys conducted before 1 January 1985 were excluded. This cut-off point was informed by many reports of the changing patterns of malaria epidemiology coincidental with this period [11-28].
Sample size	A sample size of 50 was deemed an adequate minimum based on the inverse relationship observed between sample size and the standard error of prevalence estimates [10].
Sampling method	Surveys needed to be community based and conducted in the whole community or in a random sample of the entire population. Surveys of symptomatic individuals or those focused on sub-groups of the population (*e.g.* pregnant women or workers) were excluded as the former are likely to overestimate the PR and the latter are not necessarily a good representation of the entire community.
Data from intervention studies	Data from surveys conducted as part of intervention trials were included only as those representing pre-intervention baseline characterisations. Exceptions were made by using control groups only when no

	placebo or non-modifying placebos were given.
Spatial and time duplicates (*i.e.* same communities with two different PR estimates in time)	PR data from locations surveyed within a time space of 36 months were collapsed into a single estimate. If more than one PR estimate were available for a single site expanding in time beyond the 36 month window, the most recent data were kept, unless major advantages in the quality of the older data were apparent (*e.g.* significantly larger [>10%] sample size, a more appropriate age-range, or more cross-sectional observations were conducted).
Preferred criteria	
Numerator/denominator	The numerator and denominator needed to be specified clearly or derived unambiguously from the data presented. Exceptions were made only where the sample size could still be assumed safely to be above 50 (*e.g.* the source stated clearly that the whole or most of the community was sampled or that the sample size was of a number higher than 50).
Age groups	Surveys undertaken in children <14 years of age were preferred in Africa and 'all ages' elsewhere.
Spatial coverage	Since data needed to be geo-positioned with accuracy, sites or communities representing spatial points were preferred.
Examination method	Given its ubiquity as a means for malaria diagnosis, the preferred parasite detection method was microscopy, either light or fluorescent. RDT were considered when no alternative data from blood smears were available. PCR and IFAT were excluded given differing sensitivities and inter-comparability issues.

References

1. Hay SI, Snow RW, Rogers DJ: **From predicting mosquito habitat to malaria seasons using remotely sensed data: Practice, problems and perspectives**. *Parasitol Today* 1998, **14**(8):306-313.
2. Hay SI, Snow RW, Rogers DJ: **Predicting malaria seasons in Kenya using multitemporal meteorological satellite sensor data**. *Trans R Soc Trop Med Hyg* 1998, **92**(1):12-20.
3. Molineaux L: **The epidemiology of human malaria as an explanation of its distribution, including some implications for its control**. In: *Malaria: Principles and Practice of Malariology.* Edited by Wernsdorfer WH, McGregor I, vol. 2. London: Churchill Livingstone; 1988: 913-998.
4. Mabaso ML, Craig M, Vounatsou P, Smith T: **Towards empirical description of malaria seasonality in southern Africa: the example of Zimbabwe**. *Trop Med Int Health* 2005, **10**(9):909-918.
5. Smith T, Charlwood JD, Kihonda J, Mwankusye S, Billingsley P, Meuwissen J, Lyimo E, Takken W, Teuscher T, Tanner M: **Absence of seasonal variation in malaria parasitaemia in an area of intense seasonal transmission**. *Acta Trop* 1993, **54**(1):55-72.
6. Snow RW, Schellenberg JR, Peshu N, Forster D, Newton CR, Winstanley PA, Mwangi I, Waruiru C, Warn PA, Newbold C *et al*: **Periodicity and space-time clustering of severe childhood malaria on the coast of Kenya**. *Trans R Soc Trop Med Hyg* 1993, **87**(4):386-390.
7. Druilhe P, Pradier O, Marc JP, Miltgen F, Mazier D, Parent G: **Levels of antibodies to *Plasmodium falciparum* sporozoite surface antigens reflect malaria transmission rates and are persistent in the absence of reinfection**. *Infect Immun* 1986, **53**(2):393-397.
8. Rogier C, Trape JF: **Malaria attacks in children exposed to high transmission: who is protected?** *Trans R Soc Trop Med Hyg* 1993, **87**(3):245-246.
9. Baird JK: **Host age as a determinant of naturally acquired immunity to *Plasmodium falciparum***. *Parasitol Today* 1995, **11**(3):105-111.
10. Jovani R, Tella JL: **Parasite prevalence and sample size: misconceptions and solutions**. *Trends Parasitol* 2006, **22**(5):214-218.
11. Wongsrichanalai C, Pickard AL, Wernsdorfer WH, Meshnick SR: **Epidemiology of drug-resistant malaria**. *Lancet Infect Dis* 2002, **2**(4):209-218.
12. D'Alessandro U, Buttiens H: **History and importance of antimalarial drug resistance**. *Trop Med Int Health* 2001, **6**(11):845-848.
13. Hartl DL: **The origin of malaria: mixed messages from genetic diversity**. *Nat Rev Microbiol* 2004, **2**(1):15-22.
14. WHO: **Strategy to Roll Back Malaria in the WHO European Region**. In. Copenhagen: World Health Organization Regional Office for Europe; 1999.
15. WHO: **The Use of Antimalarial Drugs: Report of an Informal Consultation**. In. Geneva: World Health Organization; 2001.
16. Wernsdorfer WH: **Epidemiology of drug resistance in malaria**. *Acta Trop* 1994, **56**(2-3):143-156.
17. Curtis CF, Lines JD: **Should DDT be banned by international treaty?** *Parasitol Today* 2000, **16**(3):119-121.
18. Curtis CF: **Should the use of DDT be revived for malaria vector control?** *Biomedica* 2002, **22**(4):455-461.

19. Hemingway J, Ranson H: **Insecticide resistance in insect vectors of human disease**. *Annu Rev Entomol* 2000, **45**:371-391.
20. Majori G, Sabatinelli G, Kondrachine AV: **Re-emerging malaria in the WHO European region: control priorities and constraints**. *Parassitologia* 1999, **41**(1-3):327-328.
21. Sabatinelli G: **The malaria situation in the WHO European region [in Russian]**. *Med Parazitol (Mosk)* 2000(2):4-8.
22. Park JW, Klein TA, Lee HC, Pacha LA, Ryu SH, Yeom JS, Moon SH, Kim TS, Chai JY, Oh MD *et al*: **Vivax malaria: a continuing health threat to the Republic of Korea**. *Am J Trop Med Hyg* 2003, **69**(2):159-167.
23. Hay SI, Cox J, Rogers DJ, Randolph SE, Stern DI, Shanks GD, Myers MF, Snow RW: **Climate change and the resurgence of malaria in the East African highlands**. *Nature* 2002, **415**(6874):905-909.
24. WHO: **Vector Resistance to Pesticides**. In: *WHO Technical Report Series.* Geneva: World Health Organization; 1992.
25. Trape JF, Pison G, Preziosi MP, Enel C, Desgrees du Lou A, Delaunay V, Samb B, Lagarde E, Molez JF, Simondon F: **Impact of chloroquine resistance on malaria mortality**. *C R Acad Sci III* 1998, **321**(8):689-697.
26. Snow RW, Trape JF, Marsh K: **The past, present and future of childhood malaria mortality in Africa**. *Trends Parasitol* 2001, **17**(12):593-597.
27. Shanks GD, Biomndo K, Hay SI, Snow RW: **Changing patterns of clinical malaria since 1965 among a tea estate population located in the Kenyan highlands**. *Trans R Soc Trop Med Hyg* 2000, **94**(3):253-255.
28. Talisuna AO, Bloland P, D'Alessandro U: **History, dynamics, and public health importance of malaria parasite resistance**. *Clin Microbiol Rev* 2004, **17**(1):235-254.
